# Supplementary material for: Mycobacterial HelD connects RNA polymerase recycling with transcription initiation
Source: Nat Commun. 2024 Oct 9;15:8740. doi: 10.1038/s41467-024-52891-5 (PMC11464796; doi:10.1038/s41467-024-52891-5)
Supplement: Supplementary file 1 — Supplementary Information [file 41467_2024_52891_MOESM1_ESM.pdf]

Supplementary information: Mycobacterial HelD connects RNA polymerase recycling with transcription initiation

Tomáš Koval'[1*], Nabajyoti Borah[2,3*], Petra Sudzinová[2], Barbora Brezovská[2], Hana Šanderová[2], Viola Vaňková Hausnerová[2,3], Alena Křenková[4], Martin Hubálek[4], Mária Trundová[1], Kristýna Adámková[1], Jarmila Dušková[1], Marek Schwarz[2], Jana Wiedermannová[2], Jan Dohnálek[1#], Libor Krásný[2#] and Tomáš Kouba[4#]

[1]Institute of Biotechnology of the Czech Academy of Sciences, Průmyslová 595, 252 50 Vestec, Czech Republic

[2]Institute of Microbiology of the Czech Academy of Sciences, Vídeňská 1083, 142 20 Prague, Czech Republic

[3]Department of Genetics and Microbiology, Faculty of Science, Charles University, Viničná 5, 128 44 Prague, Czech Republic

[4]Institute of Organic Chemistry and Biochemistry of the Czech Academy of Sciences, Flemingovo náměstí 542/2, 160 00 Prague, Czech Republic

*These authors contributed equally
#email: tomas.kouba@uochb.cas.cz; krasny@biomed.cas.cz; Jan.Dohnalek@ibt.cas.cz

Contents:

Supplementary Figures 1-20

Supplementary Tables 1-4

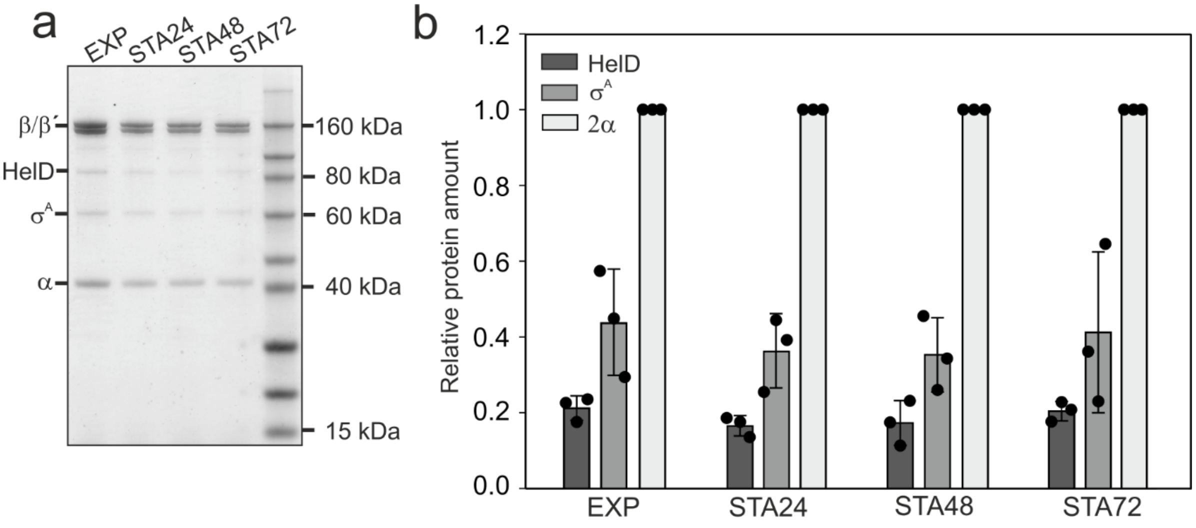

	EXP	STA24	STA48	STA72
σ^A/HelD	2.03±0.38	2.17±0.33	2.08±0.19	1.95±0.79

Supplementary Figure 1: Relative amounts of HelD and σ^A on RNAP.

Relative amounts of σ^A and HelD bound to RNAP as extracted from *Mycobacterium smegmatis (Msm)* strain LK1468 (containing FLAG on β subunit) from exponential (EXP) and three time points in stationary phase (STA after 24, 48 and 72 hours after inoculation). Subsequently, immunoprecipitations using anti-FLAG antibody were performed and analyzed on Coomassie blue-stained SDS-PAGE.

a, Representative SDS-PAGE. Molecular weight marker is indicated on the right. Source data are provided as the Source Data file.

b, Quantitation of the data by densitometry. The amount of the α subunit dimer was set as 1. The bars show averages from three biological replicas. The bars show relative stochiometric amounts of the selected proteins compared to the α dimer. Error bars show ± SD. Numerical values of averages of the ratio of σ^A/HelD amounts ± SD are in the table below the graph. Source data are provided as the Source Data file.

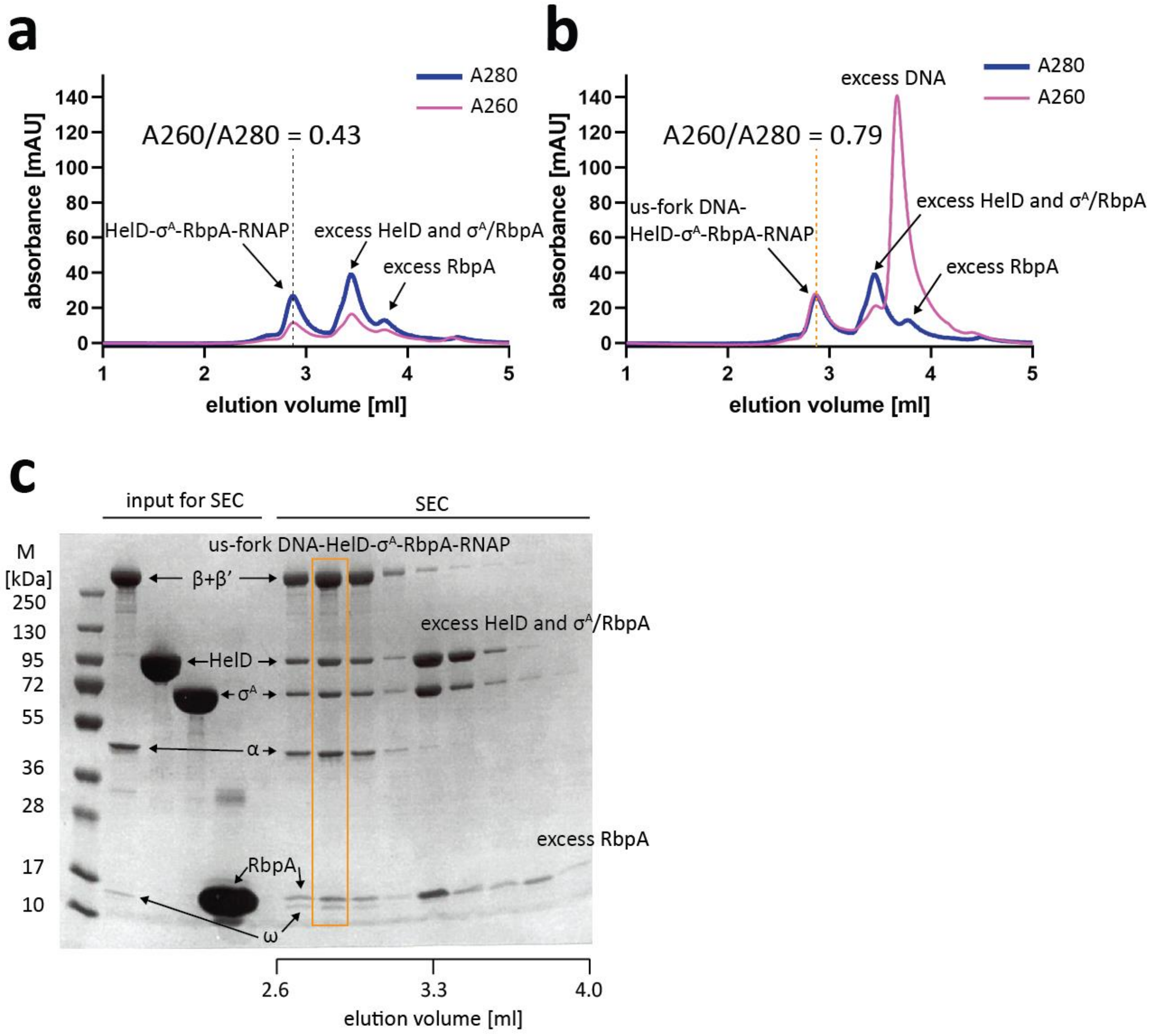

Supplementary Figure 2: Size exclusion chromatography (SEC) analysis of *Msm* RNAP core together with σ^A, RbpA, HelD and with and without us-fork DNA.

a, Reconstitution of RNAP core with excess of HelD, σ^A, and RbpA, and subsequent SEC. Absorbance at 260 and 280 nm is marked in purple and blue, respectively.

b, Reconstitution of RNAP core with the excess of HelD, σ^A, and RbpA and us-fork DNA, and subsequent SEC. Absorbance at 260 and 280 nm is marked in purple and blue, respectively.

c, SDS-PAGE analysis of SEC in panel **b**. The orange box highlights the fraction corresponding to the complex peak in panel **b**. The Mw marker used was PageRuler (Thermo Scientific).

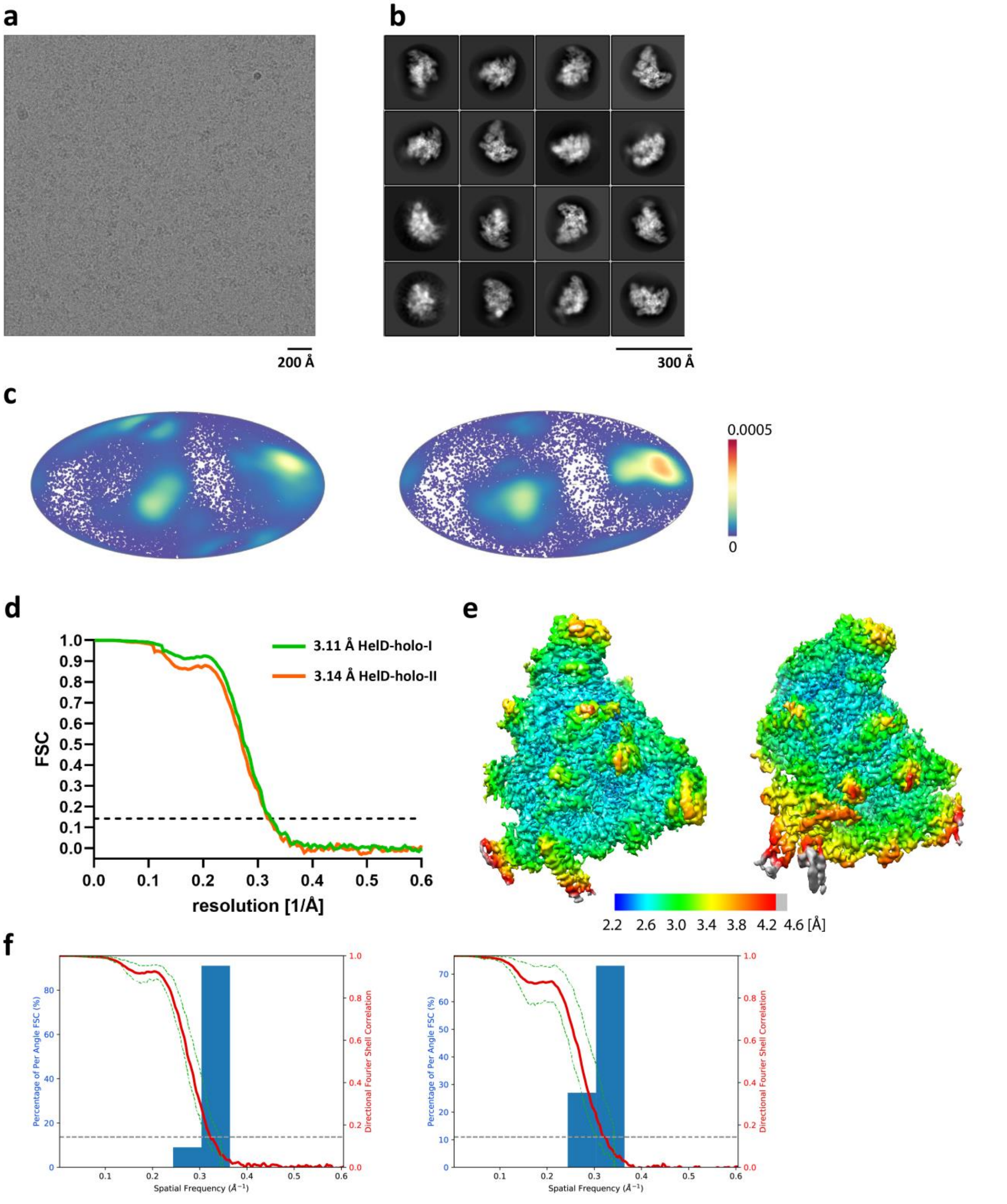

Supplementary Figure 3: Cryo-EM of the *Msm* HelD-σ^A-RbpA-RNAP State I (HelD-holo-I) and HelD-σ^A-RbpA-RNAP State II (HelD-holo-II) complexes.

a, Micrograph of the H-σ-I and -II complexes in free standing ice after MotionCor2[1] correction at defocus of ~2.5 µm.

b, 2D-class averages of the HelD-holo-I and -II complexes.

c, Angular distribution for particles of the HelD-holo-I (**left**) and -II (**right**) complexes on globe-like plane. Every point is a particle orientation and the color scale represents the normalized density of views around this point. The color scale runs from 0 (low, blue) to 0.0005 (high, red).

d, Fourier shell correlation (FSC) curves for the HelD-holo-I (green) and -II (orange) complexes. The plot of the FSC between two independently refined half-maps shows the overall resolution of the two maps as indicated by the gold standard FSC 0.143 cut-off criterion[2].

e, Surface representation of local resolution distribution of the HelD-holo-I (**left**) and -II (**right**) complexes. The map is colored according to the local resolution calculated within the RELION software package. Resolution is as indicated in the color bar.

f, Plots of the global half-map FSC (solid red line, right axis) together with the spread of directional resolution values defined by $\pm1\sigma$ from the mean (area encompassed by dotted green lines) and a histogram of directional FSC (blue bars, left axis) of the HelD-holo-I (**left**) and -II (**right**) complexes. Analysis was performed with the 3D FSC algorithm version 3.0[3].

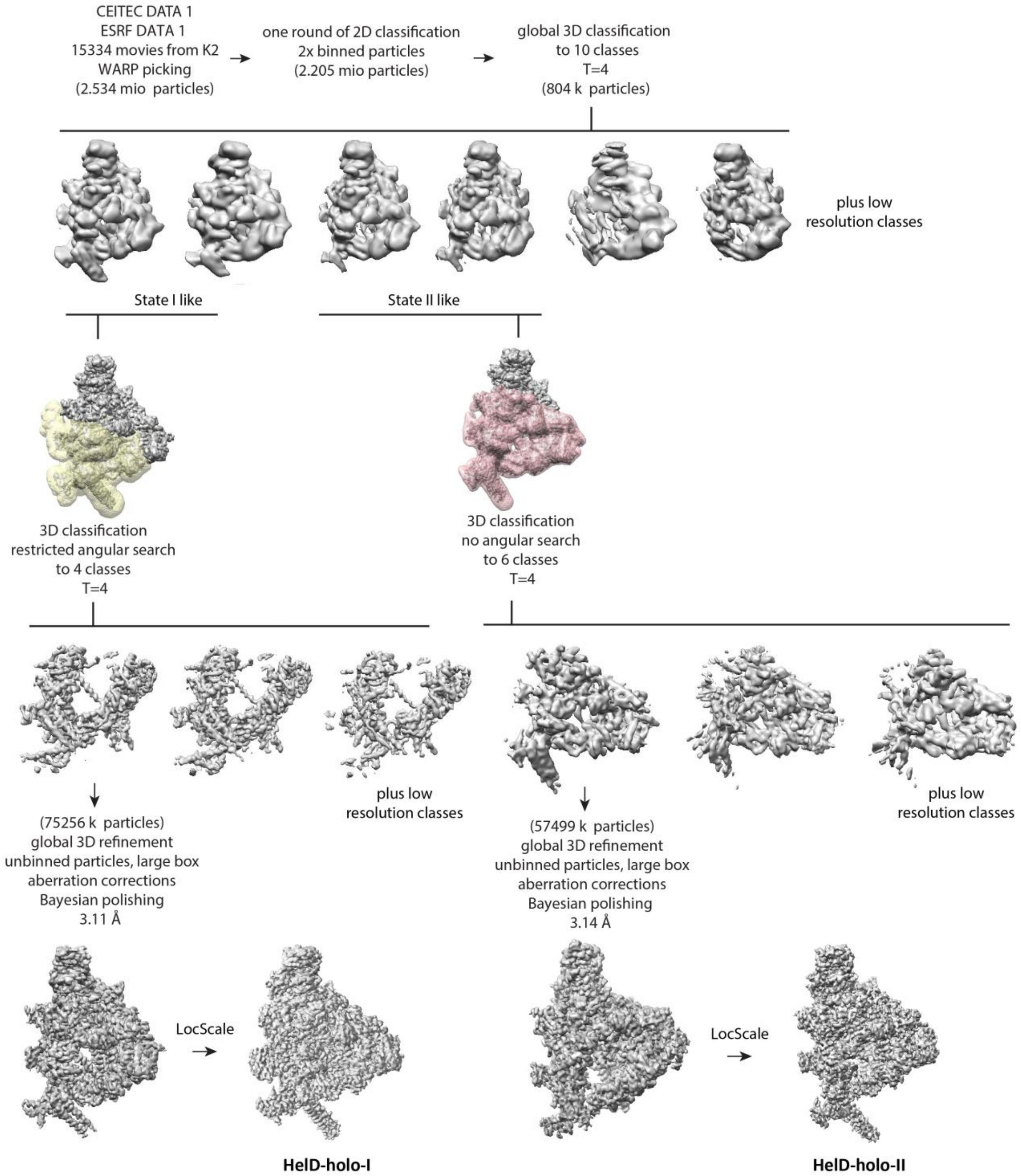

Supplementary Figure 4: Cryo-EM data 3D classification and refinement scheme of the *Msm* HelD-σ^A-RbpA-RNAP State I (HelD-holo-I) and HelD-σ^A-RbpA-RNAP State II (HelD-holo-II) complexes.

Summary of the cryo-EM 3D classification and refinement scheme of the HelD-holo-I and -II complexes. Final cryo-EM map was refined and post-processed with a respective mask in RELION 4.0[4] and filtered by LocScale[5].

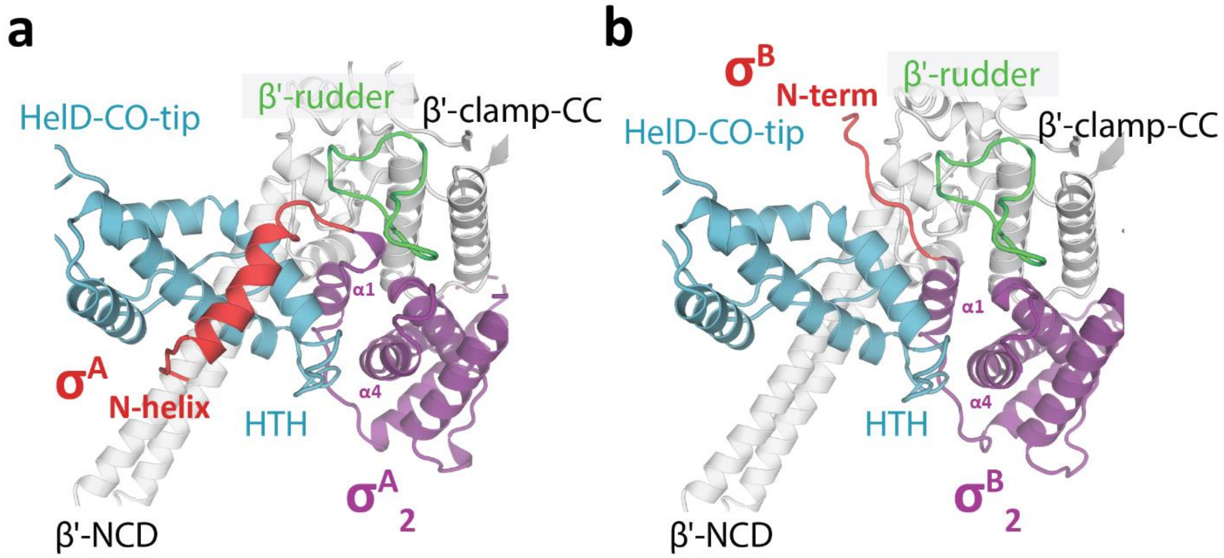

Supplementary Figure 5: σ^B is compatible with HelD binding in the context of RNAP β′-clamp.

a, Mutual interaction of σ^A and HelD in the context of the β′-clamp in the HelD-holo-II complex.

b, Superposition of *Mycobacterium tuberculosis (Mtb)* σ^B (PDB 7PP4) to the HelD-holo-II complex on the basis of *Msm* σ$^A_2$ domain. *Mtb* σ^B is visualized instead of *Msm* σ^A. The helices 1 and 4 of σ$^B_2$ are compatible with HelD CO-domain tip interaction. σ^B is missing an equivalent of the σ$^A_{N\text{-helix}}$ (**a**, shown in red), instead, a short peptide of σ$^B_{N\text{-term}}$ (residues 17-25) is folded around the β′-rudder and β′-clamp in the *Mtb* σ^B complex without HelD.

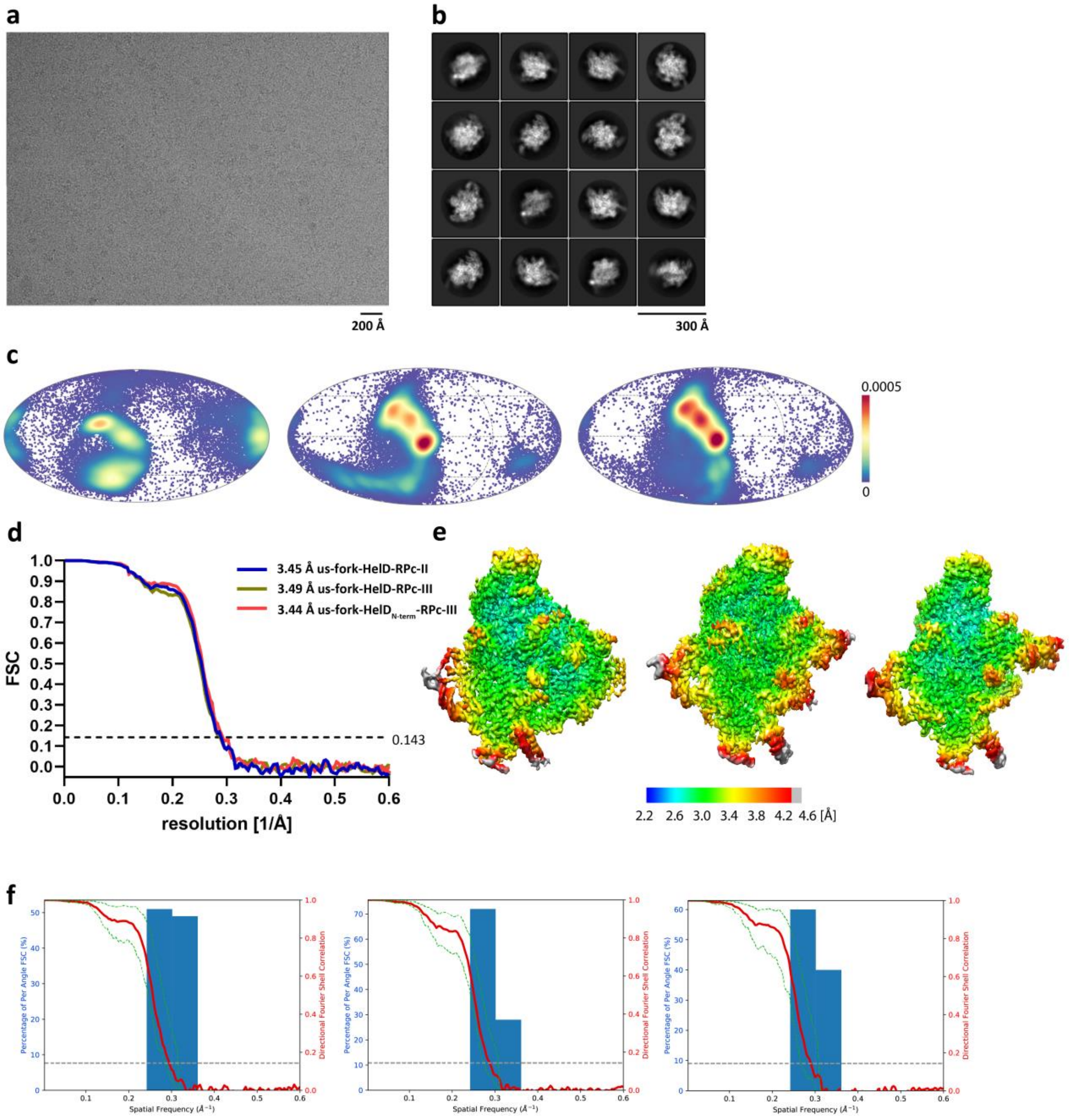

Supplementary Figure 6: Cryo-EM of the *Msm* us-fork promoter-HelD-σ^A-RbpA-RNAP State II (us-fork-HelD-RPc-II), us-fork promoter-HelD-σ^A-RbpA-RNAP State III (us-fork-HelD-RPc-III) and us-fork promoter-HelD$_{N\text{-term}}$-σ^A-RbpA-RNAP State III (us-fork-HelD$_{N\text{-term}}$-RPc-III) complexes.

a, Micrograph of the us-fork-HelD-RPc-II and -III, and us-fork-HelD$_{N\text{-term}}$-RPc-III complexes in free standing ice after MotionCor2[1] correction at defocus of ~2.5 µm.

b, 2D-class averages of the us-fork-HelD-RPc-II and -III, and us-fork-HelD$_{N\text{-term}}$-RPc-III complexes.

c, Angular distribution of particle projections of the us-fork-HelD-RPc-II (**left**) and -III (**middle**), and us-fork-HelD$_{N\text{-term}}$-RPc-III (**right**) complexes on a globe-like plane. Every point is a particle

orientation and the color scale represents the normalized density of views around this point. The color scale runs from 0 (low, blue) to 0.0005 (high, red).

d, Fourier shell correlation (FSC) curves for the us-fork-HelD-RPc-II (red) and -III (blue), and us-fork-HelD$_{N\text{-}term}$-RPc-III (dark green) complexes. The plot of FSC between two independently refined half-maps shows the overall resolution of the two maps as indicated by the gold standard FSC 0.143 cut-off criterion[2].

e, Surface representation of local resolution distribution of the us-fork-HelD-RPc-II (**left**) and -III (**middle**), and us-fork-HelD$_{N\text{-}term}$-RPc-III (**right**) complexes. The map is colored according to the local resolution calculated within the RELION software package. Resolution is as indicated in the color bar.

f, Plots of the global half-map FSC (solid red line, right axis) together with the spread of directional resolution values defined by ±1σ from the mean (area encompassed by dotted green lines) and a histogram of directional FSC (blue bars, left axis) of the us-fork-HelD-RPc-II (**left**) and -III (**middle**), and us-fork-HelD$_{N\text{-}term}$-RPc-III (**right**) complexes. Analysis was performed with the 3D FSC algorithm version 3.0[3].

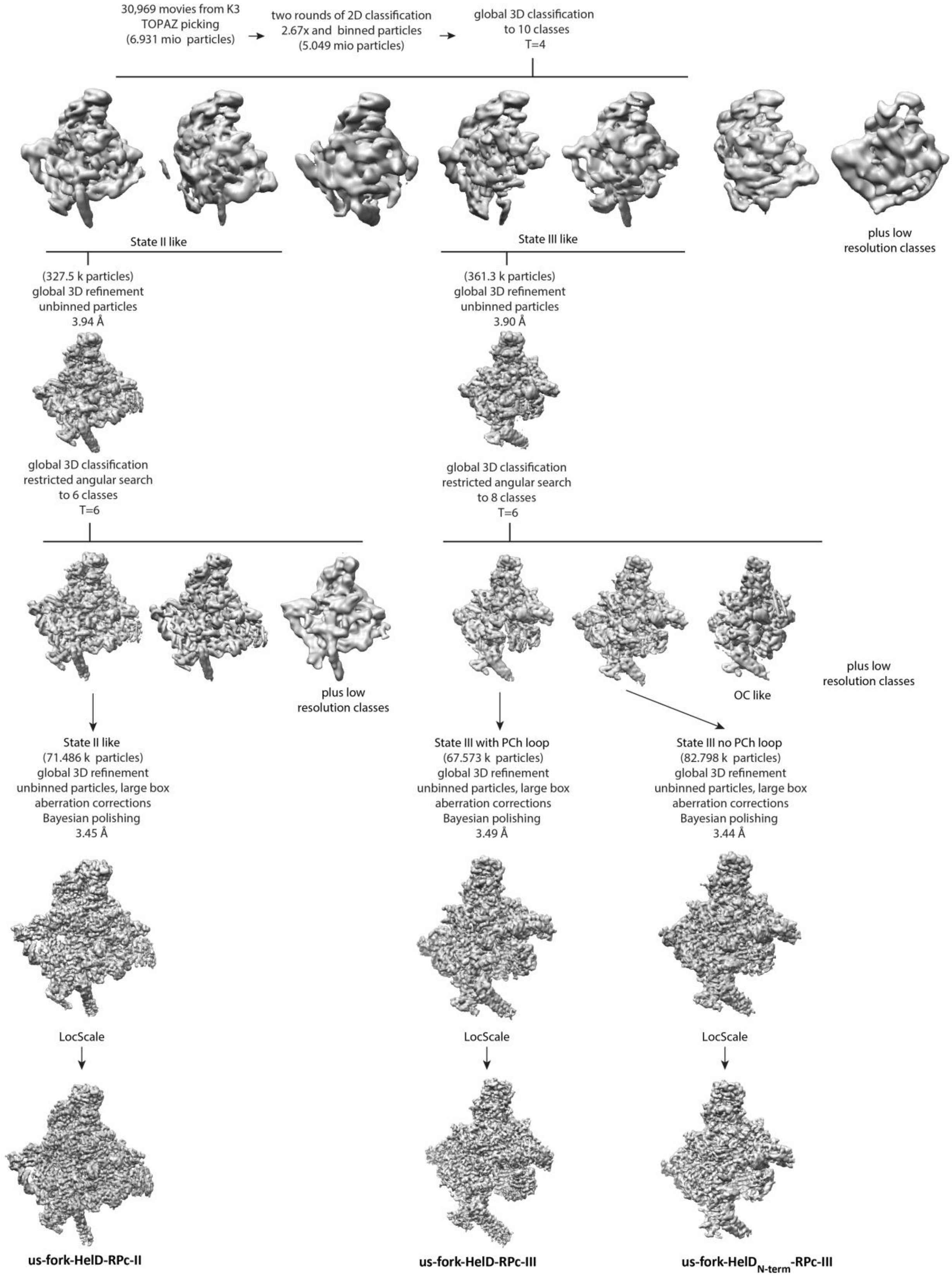
30,969 movies from K3
TOPAZ picking
(6.931 mio particles)
two rounds of 2D classification
2.67x and binned particles
(5.049 mio particles)
global 3D classification
to 10 classes
T=4
State II like
State III like
plus low
resolution classes
(327.5 k particles)
global 3D refinement
unbinned particles
3.94 Å
(361.3 k particles)
global 3D refinement
unbinned particles
3.90 Å
global 3D classification
restricted angular search
to 6 classes
T=6
global 3D classification
restricted angular search
to 8 classes
T=6
plus low
resolution classes
OC like
plus low
resolution classes
State II like
(71.486 k particles)
global 3D refinement
unbinned particles, large box
aberration corrections
Bayesian polishing
3.45 Å
State III with PCh loop
(67.573 k particles)
global 3D refinement
unbinned particles, large box
aberration corrections
Bayesian polishing
3.49 Å
State III no PCh loop
(82.798 k particles)
global 3D refinement
unbinned particles, large box
aberration corrections
Bayesian polishing
3.44 Å
LocScale
LocScale
LocScale
us-fork-HelD-RPc-II
us-fork-HelD-RPc-III
us-fork-HelD$_{N-term}$-RPc-III

Supplementary Figure 7: Cryo-EM data 3D classification and refinement scheme of the *Msm* us-fork promoter-HelD-σ^A-RbpA-RNAP State II (us-fork-HelD-RPc-II), us-fork promoter-HelD-σ^A-RbpA-RNAP State III (us-fork-HelD-RPc-III) and us-fork promoter-HelD$_{N\text{-}term}$-σ^A-RbpA-RNAP State III (us-fork-HelD$_{N\text{-}term}$-RPc-III) complexes.

Summary of the cryo-EM 3D classification and refinement scheme of the us-fork-HelD-RPc-II, -III and us-fork-HelD$_{N\text{-}term}$-RPc-III complexes. Final cryo-EM map was refined and post-processed with a respective mask in RELION 4.0[4] and filtered by LocScale[5].

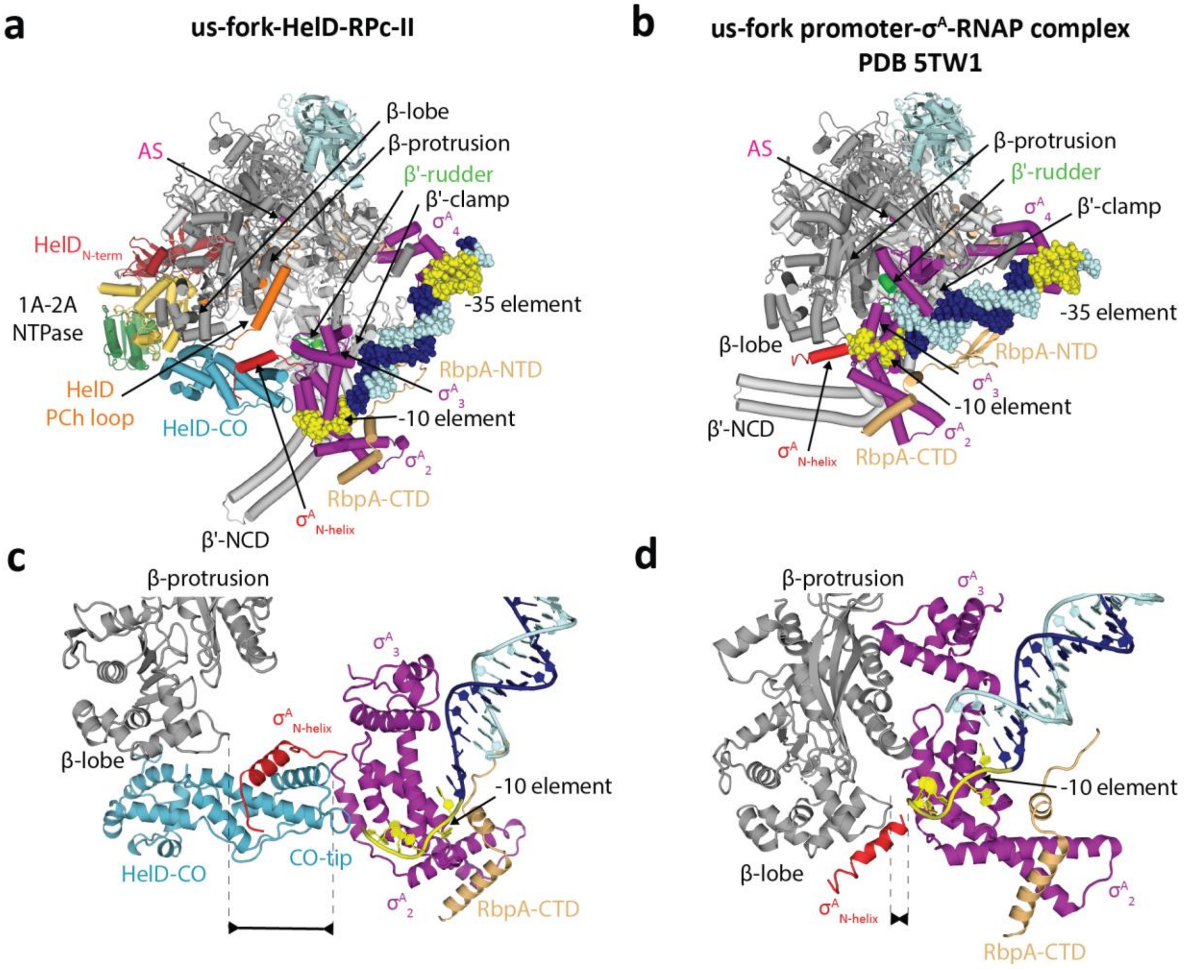

Supplementary Figure 8: Comparison of the us-fork-HelD-RPc-II and *Msm* us-fork-RbpA-$σ^A$-RNAP complex PDB 5TW1.

a, *Msm* RNAP core complex together with us-fork promoter DNA fragment, $σ^A$, RbpA and HelD in state II conformation (us-fork-HelD-RPc-II).

b, *Msm* us-fork-RbpA-$σ^A$-RNAP complex PDB 5TW1. Individual domains are color-coded according to the legend in Figure 1d.

c, and d Close-up views of the RNAP primary channel, corresponding to panels **a** and **b,** respectively. In us-fork-RbpA-$σ^A$-RNAP, complete closure of RNAP primary channel allows $σ^A_{N\text{-}helix}$ to directly interact with the β-lobe domain.

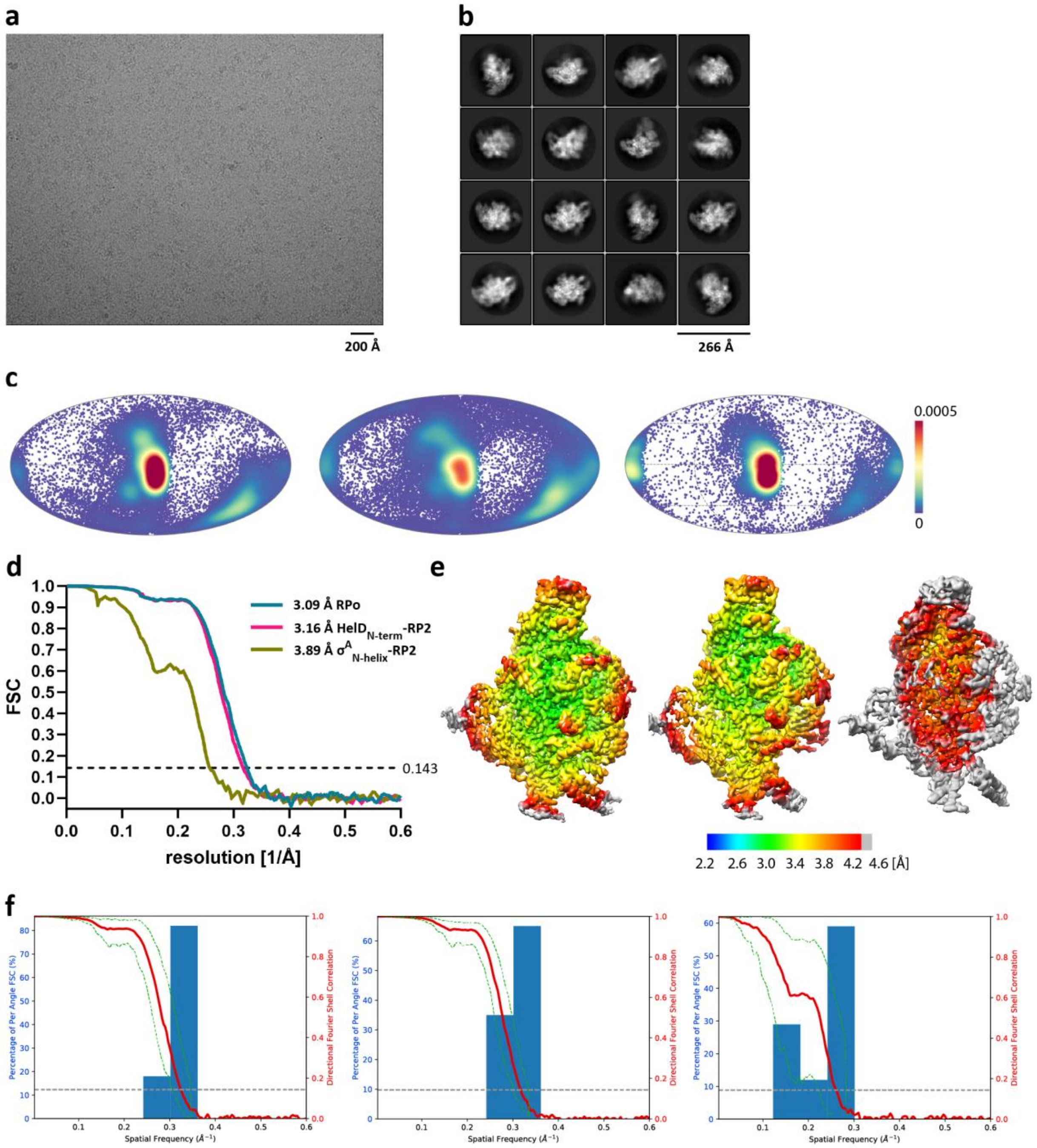

Supplementary Figure 9: Cryo-EM of the *Msm* open complex (RPo), HelD$_{\text{N-term}}$-RP2and σ$^{A}_{\text{N-helix}}$-RP2 complexes.

a, Micrograph of the RPo, HelD$_{\text{N-term}}$-RP2 and σ$^{A}_{\text{N-helix}}$-RP2 complexes in free standing ice after MotionCor2[1] correction at defocus of ~2.5 µm.

b, 2D-class averages of the RPo, HelD$_{\text{N-term}}$-RP2 and σ$^{A}_{\text{N-helix}}$-RP2 complexes

c, Angular distribution of particle projections of the RPo (**left**), HelD$_{\text{N-term}}$-RP2 (**middle**), and σ$^{A}_{\text{N-helix}}$-RP2 (**right**) complexes on a globe-like plane. Every point is a particle orientation and the color

scale represents the normalized density of views around this point. The color scale runs from 0 (low, blue) to 0.0005 (high, red).

d, Fourier shell correlation (FSC) curves for the RPo (blue), $HelD_{N\text{-}term}$-RP2 (cyan), and $\sigma^{A}_{N\text{-}helix}$-RP2 (green) complexes. The plot of the FSC between two independently refined half-maps shows the overall resolution of the two maps as indicated by the gold standard FSC 0.143 cut-off criterion.

e, Surface representation of local resolution distribution of the RPo (**left**), $HelD_{N\text{-}term}$-RP2 (**middle**), and $\sigma^{A}_{N\text{-}helix}$-RP2 (**right**) complexes. The map is colored according to the local resolution calculated within the RELION software package. Resolution is as indicated in the color bar.

f, Plots of the global half-map FSC (solid red line, right axis) together with the spread of directional resolution values defined by ±1σ from the mean (area encompassed by dotted green lines) and a histogram of directional FSC (blue bars, left axis) of the RPo (**left**), $HelD_{N\text{-}term}$-RP2 (**middle**), and $\sigma^{A}_{N\text{-}helix}$-RP2 (**right**) complexes. Analysis was performed with the 3D FSC algorithm version 3.0[3].

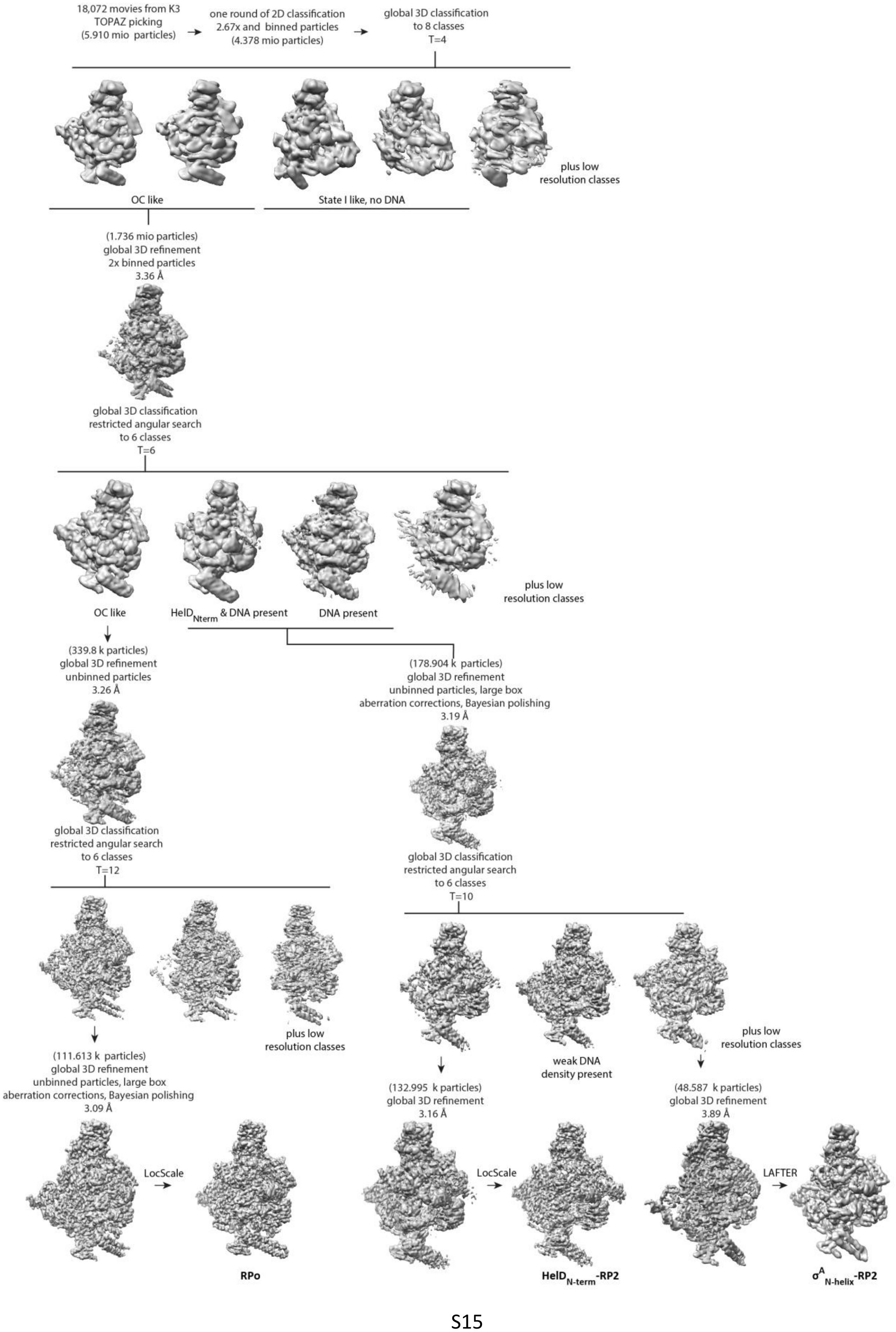
18,072 movies from K3
TOPAZ picking
(5.910 mio particles)
one round of 2D classification
2.67x and binned particles
(4.378 mio particles)
global 3D classification
to 8 classes
T=4
plus low
resolution classes
OC like
State I like, no DNA
(1.736 mio particles)
global 3D refinement
2x binned particles
3.36 Å
global 3D classification
restricted angular search
to 6 classes
T=6
plus low
resolution classes
OC like
HelD Nterm & DNA present
DNA present
(339.8 k particles)
global 3D refinement
unbinned particles
3.26 Å
(178.904 k particles)
global 3D refinement
unbinned particles, large box
aberration corrections, Bayesian polishing
3.19 Å
global 3D classification
restricted angular search
to 6 classes
T=12
global 3D classification
restricted angular search
to 6 classes
T=10
plus low
resolution classes
plus low
resolution classes
(111.613 k particles)
global 3D refinement
unbinned particles, large box
aberration corrections, Bayesian polishing
3.09 Å
weak DNA
density present
(132.995 k particles)
global 3D refinement
3.16 Å
(48.587 k particles)
global 3D refinement
3.89 Å
LocScale
LocScale
LAFTER
RPo
HelD N-term-RP2
σA N-helix-RP2

Supplementary Figure 10: Cryo-EM data 3D classification and refinement scheme of the *Msm* open complex (RPo), HelD$_{N\text{-term}}$-RP2 and $\sigma^{A}_{N\text{-helix}}$-RP2 complexes.

Summary of the cryo-EM 3D classification and refinement scheme of the RPo, HelD$_{N\text{-term}}$-RP2 and $\sigma^{A}_{N\text{-helix}}$-RP2 complexes. Final cryo-EM map was refined and post-processed with a respective mask in RELION 4.0[4]and filtered by LocScale[5] or LAFTER[6]

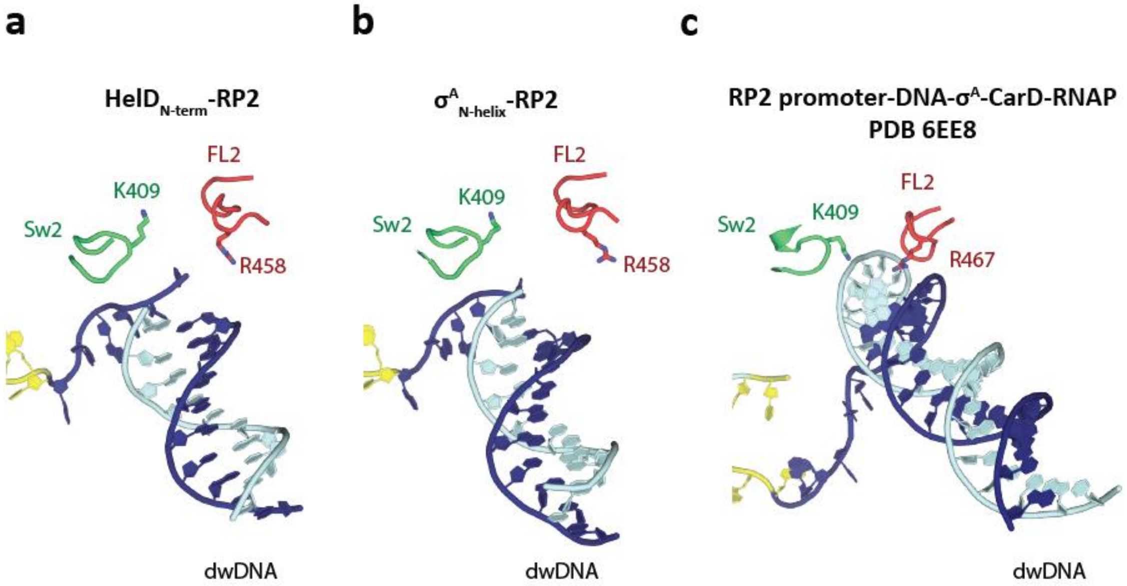

Supplementary Figure 11: Fork-loop 2 (FL2) and switch 2 (Sw2) are not engaged with the dwDNA in HelD$_{N\text{-}term}$-RP2 and $\sigma^A_{N\text{-}helix}$-RP2.

a, b, c dwDNA and the FL2 and Sw2 features in the HelD$_{N\text{-}term}$-RP2, $\sigma^A_{N\text{-}helix}$-RP2 and *Mtb* CarD RP2 (PDB 6EE8), respectively. In CarD RP2 (**c**) the dwDNA interacts with K409 of Sw2 and R467 of FL2. The equivalent residues in *Msm* (K409 and R458) are not engaged with the dwDNA in HelD$_{N\text{-}term}$-RP2 (**a**) and $\sigma^A_{N\text{-}helix}$-RP2 (**b**).

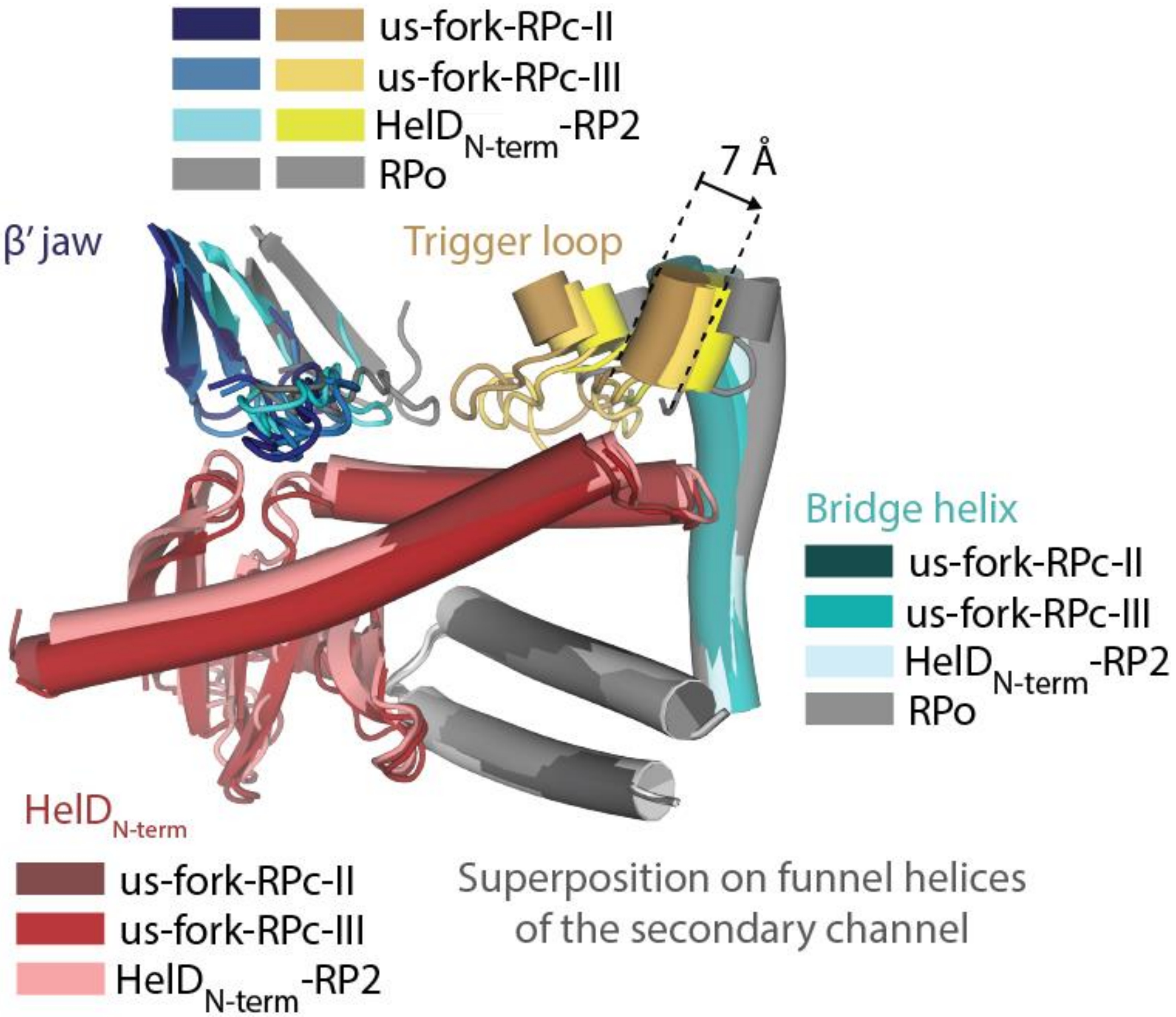

Supplementary Figure 12: Progressive conformational changes in the RNAP secondary channel upon RNAP closing lead to suboptimal binding site for HelD$_{N\text{-term}}$.

Us-fork-HelD-RPc-II (dark colors), us-fork-HelD-RPc-III (medium colors), HelD$_{N\text{-term}}$-RP2 (light colors) and open complex (grey) are shown superimposed on β′ funnel helices (residues 739-792) which are forming the constant side of the secondary channel. Upon closing of RNAP through the represented states, interactions of HelD$_{N\text{-term}}$ with the constant part of the secondary channel remain unchanged while the β′ jaw (shades of blue) and the trigger loop region of β′ (shades of yellow) shift (black arrow) toward the RNAP active site and the bridge helix (shades of cyan). The loss of contacts between HelD and β′ in this area helps disengage HelD$_{N\text{-term}}$ from RNAP.

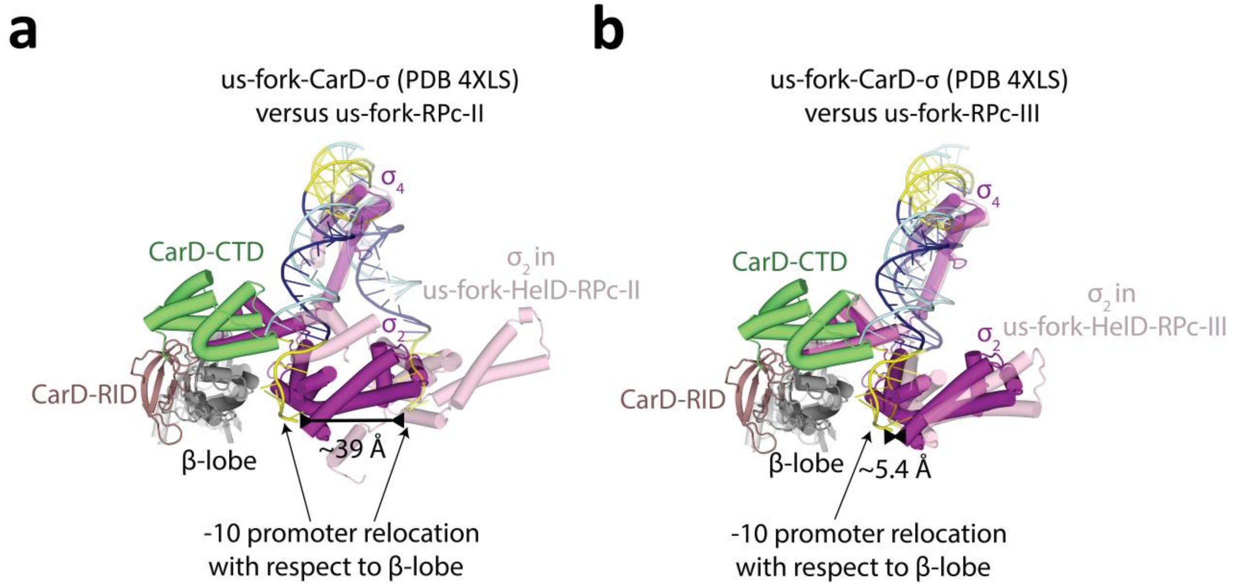

Supplementary Figure 13: CarD-CTD cannot reach to bind the −10 element when HelD is present.

a, Superposition of the us-fork-HelD-RPc-II complex (pale colors) with crystal structure of *T. aquaticus* transcription initiation complex with CarD containing upstream fork promoter (us-fork-CarD-σ; PDB 4XLS, bright colors) on the basis of the β-core region. The CarD-RID (violet) binds to the β-lobe domain (grey). The CarD-CTD (lime) wedges into the minor groove of the −10 element (yellow) in order to stabilize interaction with the promoter DNA in the context of the σ_2 subunit (purple). The superposition shows, that in the us-fork-HelD-RPc-II the −10 element together with the σ_2 are relocated by ~39 Å away from the β-lobe in comparison to us-fork-CarD-σ. This is too far for CarD to simultaneously reach both the β-lobe and −10 element biding sites.

b, Superposition of the us-fork-HelD-RPc-III (pale colors) with us-fork-CarD-σ (PDB 4XLS, bright colors). The superposition shows, that the −10 element together with σ_2 are relocated by ~5.4 Å away from the β-lobe. This is too far for CarD to simultaneously reach both the β-lobe and the −10 element.

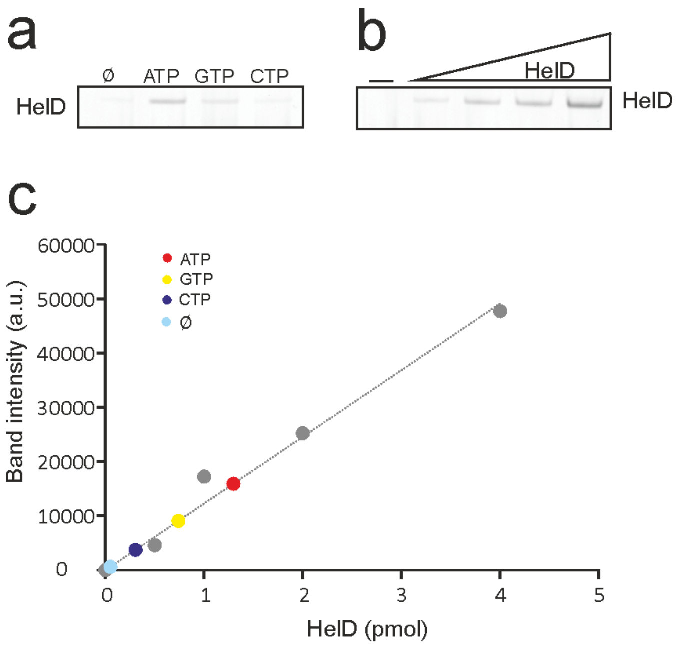

Supplementary Figure 14: An example of the quantitative densitometric analysis.

a, The gel strip shows a representative Coomassie blue-stained SDS-PAGE of NTP-induced HelD release from the RNAP-σ^A-RbpA-HelD complex (see Figure 4).

b, The gel strip shows calibration standards - serial (two-fold) dilution of HelD (0, 0.5, 1, 2, 4 pmol). Source data are provided as the Source Data file.

c, Volumetric band intensity (background subtracted) of HelD bands was obtained by quantifying increasing amounts of HelD and was plotted against HelD amounts (grey dots) to create the calibration curve. The dotted line shows the calibration curve. The spontaneous (Ø) and NTP-induced (color-coded) HelD release are indicated along the calibration curve (ATP, red dot; GTP, yellow dot; CTP, blue dot; spontaneous release, light blue dot). Source data are provided as the Source Data file.

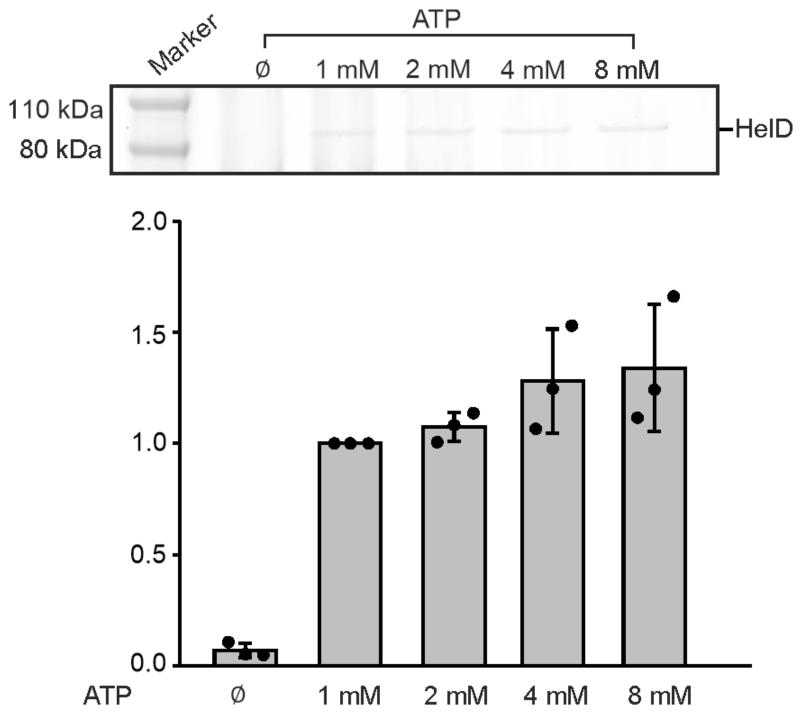

Supplementary Figure 15: Effect of increasing concentration of ATP on HelD release from RNAP.

Complexes containing His-RNAP, HelD, σ^A and RbpA were reconstituted as depicted in Figure 4c and described in detail in Methods. Subsequently, different concentrations of ATP (1 mM, 2 mM, 4 mM, 8 mM) were added to the preformed RNAP complexes attached to magnetic beads. Release of HelD from the complex to the supernatant was determined with Coomassie blue-stained SDS–PAGE gels. The primary data strip above the graph shows a representative result. The amount of HelD released by the addition of 1 mM ATP was set as 1. The bars show averages from three independent experiments and the error bars indicate ± SD. Source data are provided as the Source Data file.

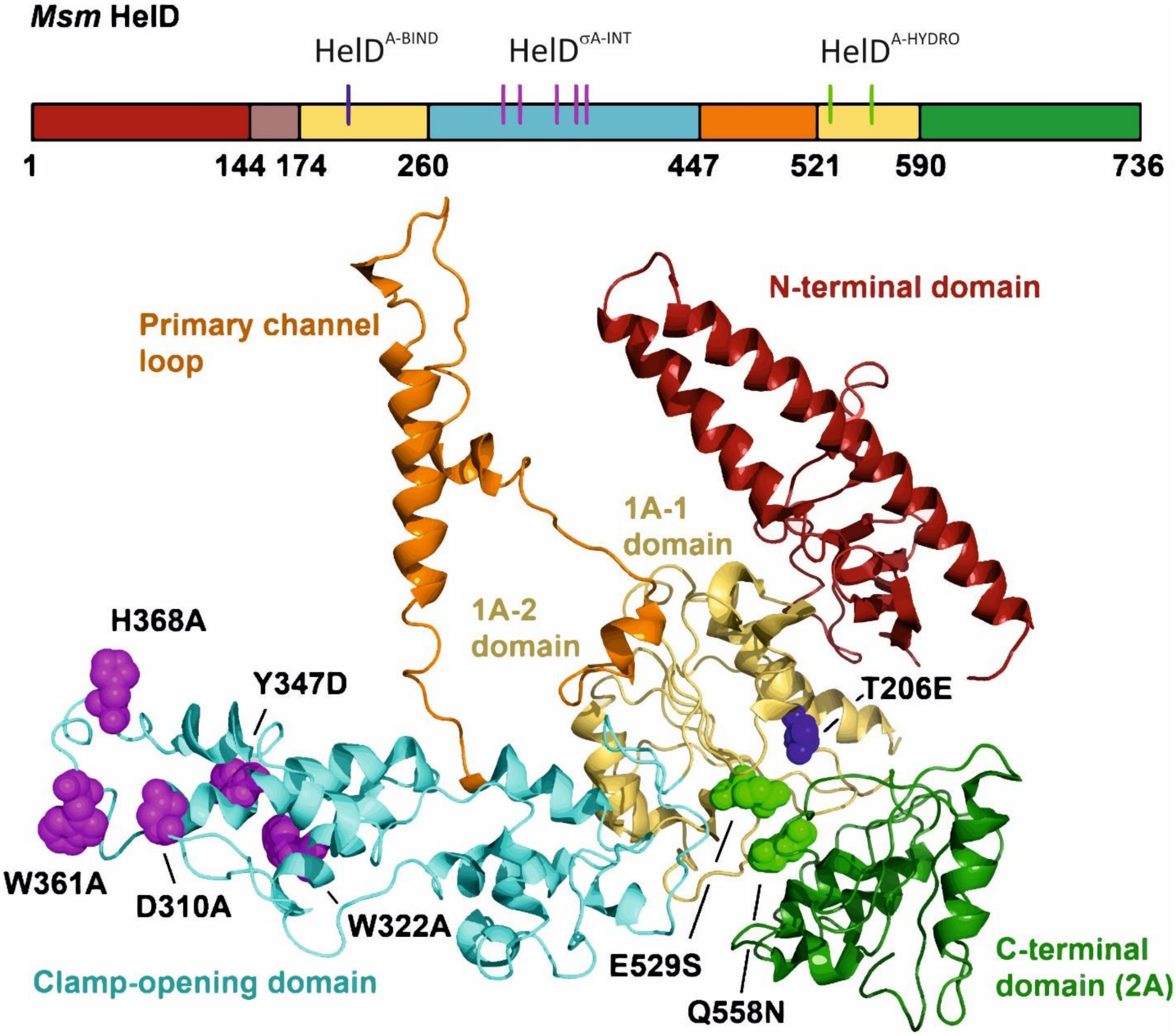

Supplementary Figure 16: A scheme showing mutations in HelD.

Positions of mutations in particular HelD mutants are marked in the schematic linear representation (upper part of the figure) and shown in the HelD structure (lower part, PDB ID: 6YYS). In the structure, mutations are shown as spheres: magenta for HelD$^{\sigma A\text{-}INT}$, light green for HelD$^{A-HYDRO}$ and dark blue for HelD$^{A\text{-}BIND}$. HelD is shown using its secondary structure elements with its N-terminal domain red, 1A-1 and 1A-2 domain yellow, primary channel loop orange, clamp-opening domain cyan and C-terminal domain green.

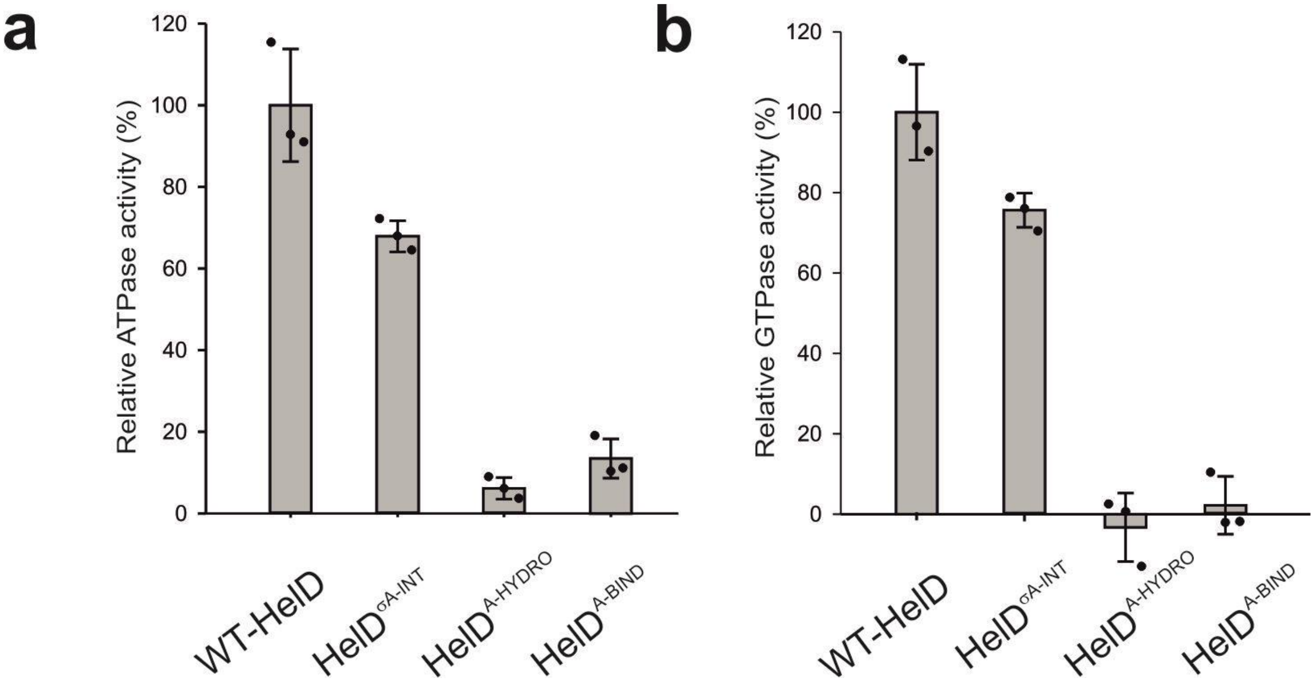

Supplementary Figure 17: ATPase and GTPase activity of WT-HelD and its mutant forms.

a, ATPase activities of WT-HelD and its mutant variants.

b, GTPase activities of WT-HelD and its mutant variants.

The bars show mean values, the error bars indicate standard deviation, the black circle symbols represent values from three independent replicates. Source data are provided as the Source Data file.

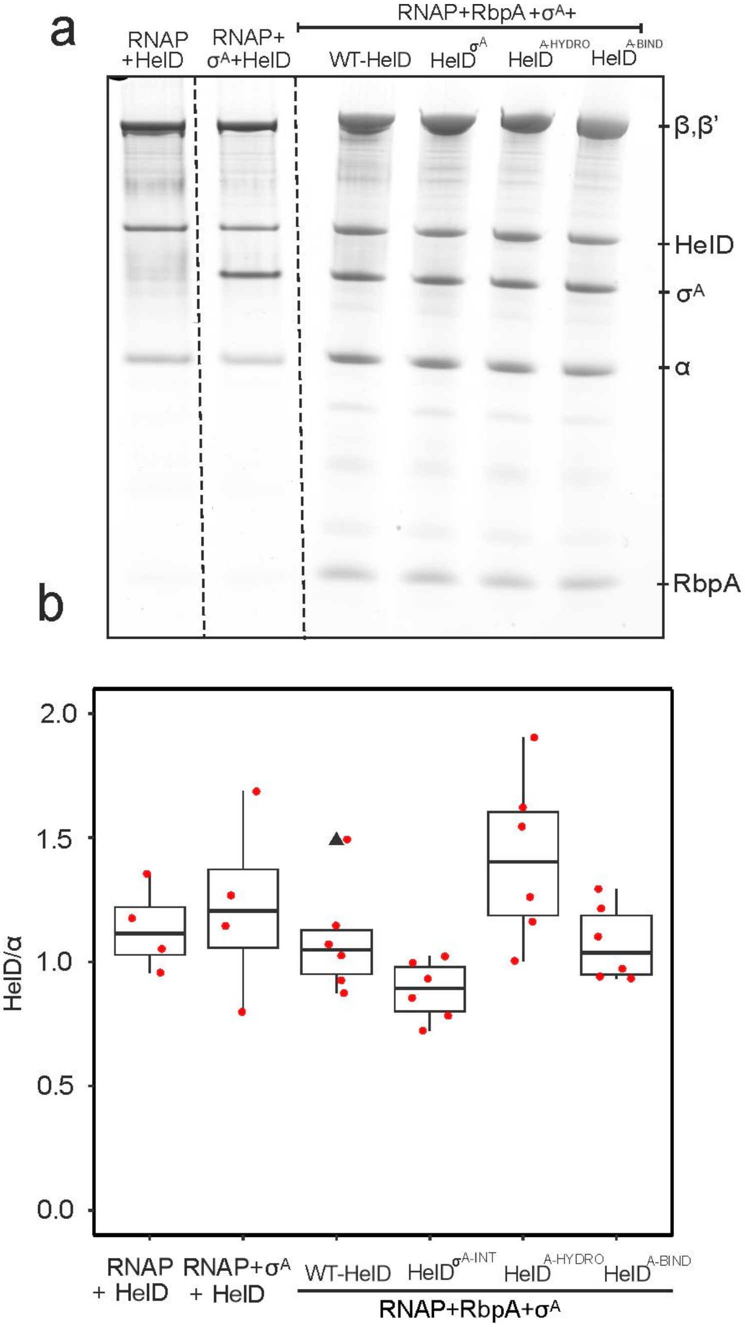

Supplementary Figure 18: Association of RNAP-HelD complexes.

a, Representative Coomassie blue-stained SDS–PAGE gel showing binding of WT and mutated HelD variants to various complexes of RNAP. Source data are provided as the Source Data file.

b, Binding efficiencies of WT and mutant HelD variants in various complexes of RNAP, RbpA, σ^A and HelD are shown relative to the amount of the α subunit of RNAP. The quantitation was done by densitometry. The figure shows ratios of bound HelD to α in per-group boxplots and as individual points (in red). The only boxplot outlier is indicated with the black triangle next to the dot. No significant differences between the relative intensities of WT-HelD and its mutated variants were detected (two-sample *t*-test, N = 6 per group). These relative band intensities were plotted with ggplot2 in R with default settings (whiskers extend to the highest (lowest) value within 1.5×IQR beyond upper (lower) quartile).

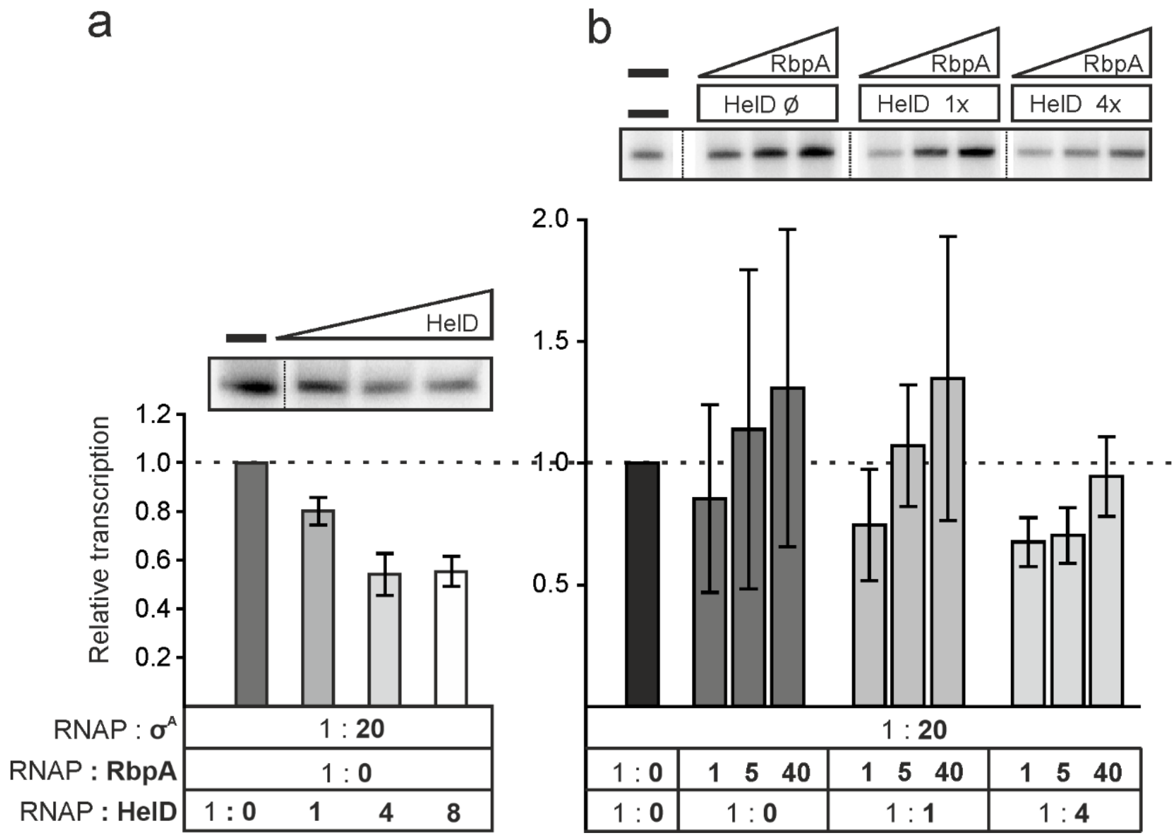

Supplementary Figure 19. Addition of HelD decreases transcription *in vitro*.

a, Multiple-round transcriptions from the *M. smegmatis* rRNA P*rrnAPCL1* promoter were performed with RNAP reconstituted with σ^A in the absence or presence of increasing amounts of HelD. Representative primary data are shown above the graphs. The dotted lines indicate where the gel was electronically assembled. The bars show averages from three independent experiments and the error bars indicate ± SD. Transcription in the absence of HelD was set as 1. Source data are provided as the Source Data file.

b, Multiple-round transcriptions were performed with RNAP reconstituted with σ^A in the presence of increasing amounts of RbpA at three different HelD concentrations (ratios are indicated below the graphs). The experiment was performed twice, the bars show averages and the error bars the range. Source data are provided as the Source Data file.

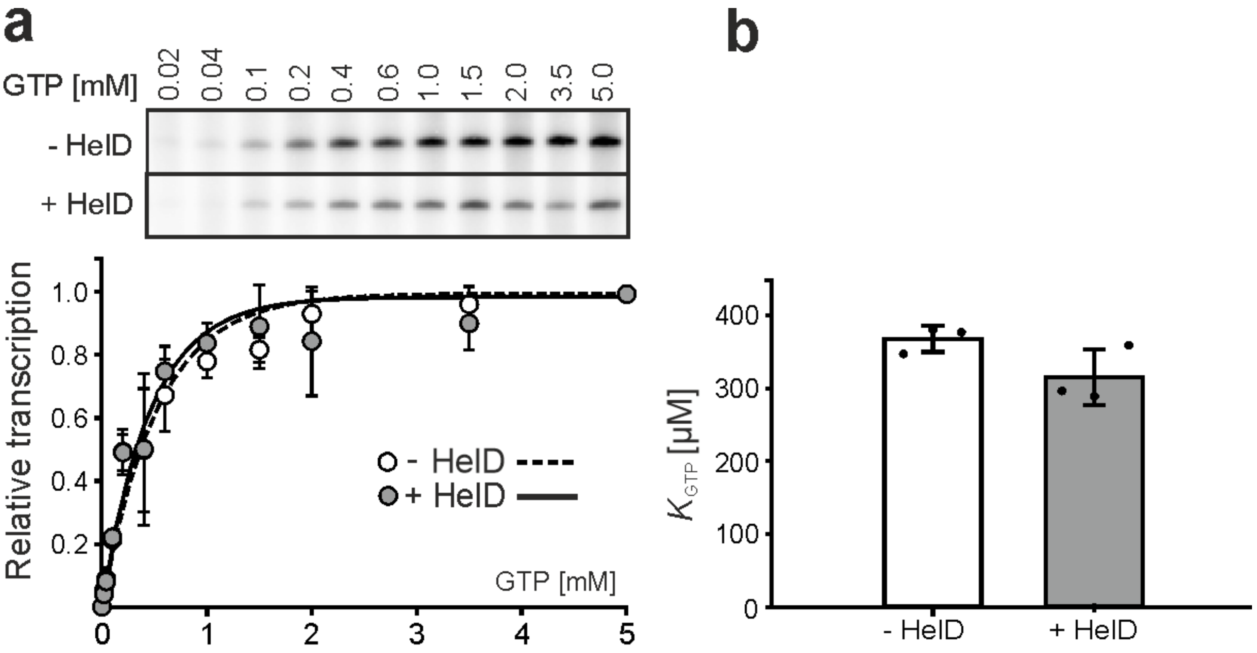

Supplementary Figure 20. Effect of HelD on the affinity of RNAP for the iNTP.

a, Multiple-round transcriptions from the *M. smegmatis* rRNA P*rrnAPCL1* promoter as a function of GTP concentration performed in the presence/absence of HelD: representative primary data are shown above the graph. The experiment was conducted in three biological replicates, the dots represent averages, and the error bars show ±SD. The primary data show 11 lanes and the plot has 12 data points. The extra data point in the plot is for [GTP] = 0, which was not tested, as it would yield zero transcription. The maximum signal from each transcription was set as 1 to facilitate comparison. Source data are provided as the Source Data file.

b, Comparison of K_{GTP} values (GTP concentrations required for half-maximal transcription) for transcription with and without HelD. The values are calculated from three experiments, the error bars show ± SD. The dots represent values from individual experiments. Source data are provided as the Source Data file.

Supplementary Table 1: Significantly enriched proteins in HelD-FLAG pull-down (Exponential phase).

Uniprot ID	Protein	Protein Enrichment *	$-\log_{10}$ p value **
P60281	DNA-directed RNA polymerase subunit β	11.74331665	3.50361185
A0QUE0	HelD	11.66900444	2.088515719
A0QW02	RNA polymerase sigma factor, σ^A	11.52702713	5.4905894
A0QS66	DNA-directed RNA polymerase subunit β′	11.3415699	3.726705787
A0QSL8	DNA-directed RNA polymerase subunit α	10.02848752	3.7668162
A0QZ11	RNA polymerase-binding protein RbpA	8.557800293	3.52823238
A0QWT1	DNA-directed RNA polymerase subunit ω	7.991231918	2.777949991
A0QVZ5	RNA polymerase sigma factor, σ^B	5.79804039	3.008321384
A0QTP7	Transcriptional regulator WhiB	5.103434245	2.318609876
A0R7G3	Uncharacterized protein	4.62999026	2.823311657
A0QXA3	Pyruvate kinase	2.821750641	2.844904942
A0R618	Acyl-CoA synthase	2.662979126	2.105244605
A0R5N8	Aspartokinase	2.220167796	2.616558561
A0QSL0	Thioredoxin reductase	2.009901047	2.944732319
A0QWV9	Probable cell division protein WhiA	1.805962245	2.224640431

* Protein enrichment is expressed as Fold change ($\log_2$).

** The abundance of individual proteins was compared by two-tailed student's t-test. The permutation-based FDR was used as an adjustment of p-value.

Supplementary Table 2: Significantly enriched proteins in HelD-FLAG pull-down (Stationary phase).

Uniprot ID	Protein	Protein Enrichment *	$-\log_{10}$ p value **
P60281	DNA-directed RNA polymerase subunit β	14.21423022	5.036702342
A0QUE0	HelD	13.97419675	4.195341446
A0QS66	DNA-directed RNA polymerase subunit β′	13.8114713	4.006223769
A0QW02	RNA polymerase sigma factor, σ^A	12.5668691	4.807812497
A0QSL8	DNA-directed RNA polymerase subunit α	11.82234828	3.366303562
A0QZ11	RNA polymerase-binding protein RbpA	9.852303823	3.56250059
A0QVZ5	RNA polymerase sigma factor, σ^B	8.511658986	4.445965436
A0QWT1	DNA-directed RNA polymerase subunit ω	7.356604258	3.273010812
A0QS98	Elongation factor Tu	4.584452311	2.492793054
A0QQU5	Chaperonin GroEL 2	4.117930094	2.604276603
A0QWT3	S-adenosylmethionine synthase	3.320119222	2.016881533
A0QSG6	Small ribosomal subunit protein uS5	3.158418655	2.174236933
A0QQC8	Chaperone protein DnaK	2.597347895	2.261128983

A0QXC0	Branched-chain amino acid ABC transporter substrate-binding protein	1.945847193	2.154263356
A0QX46	Isoleucine--tRNA ligase	1.846848806	3.034004467

***** Protein enrichment is expressed as Fold change (log_2).

****** The abundance of individual proteins was compared by two-tailed student's t-test. The permutation-based FDR was used as an adjustment of p-value.

Supplementary Table 3: Primary channel closure: distances between the β and β′ subunits at the narrowest point of the dwDNA entrance: *Msm*: β Gly 275 Cα and β′ Tyr 130 Cα; *Mtb*: β Gly 284 Cα and β′ Tyr 130 Cα.

RNAP complex	Organism	Distance (Å)	PDB entry	Reference
Holo1	*Msm*	29.4	6EYD	[7]
Holo2	*Msm*	33.6	n/a	[7]
TIC	*Msm*	14.8	5TW1	[8]
HelD-holo-I	*Msm*	34.2	8Q3I	This study
HelD-holo-II	*Msm*	43.2	8QN8	This study
us-fork-HelD-RPc-II	*Msm*	46.3	8QU6	This study
us-fork-HelD-RPc-III	*Msm*	30.6	8R3M	This study
us-fork-$HelD_{N-term}$-RPc-III	*Msm*	20.8	8R2M	This study
$HelD_{N-term}$-RP2	*Msm*	23.6	8R6P	This study
$\sigma^{A}_{N-helix}$-RP2	*Msm*	24.7	8R6R	This study
RP2	*Mtb*	19.7	6EE8	[9]
RPo	*Msm*	16.3	8QTI	This study

Supplementary Table 4: CryoEM structure determination and validation statistics.

**Name of structure**	**HelD-holo-I**	**HelD-holo-II**	**us-fork-HelD-RPc-II**	**us-fork-HelD-RPc-III**	**us-fork-HelD$_{N\text{-}term}$-RPc-III**
PDB ID	**8Q3I**	**8QN8**	**8QU6**	**8R3M**	**8R2M**
EMDB ID	**EMD-18128**	**EMD-18511**	**EMD-18656**	**EMD-18873**	**EMD-18851**
**Data collection and processing**					
Microscope	FEI Titan Krios		FEI Titan Krios		
Voltage (kV)	300		300		
Camera	Gatan K2 Summit		Gatan K3 BioQuantum		
Magnification	165,000x		105,000 x		
Nominal defocus range (µm)	1.2-3.2		0.4-2.4		
Exposure time (s)	8		2		
Electron exposure (e–/Å^2)	55		40		
Number of frames collected (no.)	40		40		
Number of frames processed (no.)	26		20		
Pixel size (Å)	0.827		0.8336		
Micrographs (no.)	15,334		30,969		
Total particle images (no.)	2,534,508		6,930,185		
**Refinement**					
Particles per class (no.)	75,256	57,499	71,486	67,573	82,798
Sphericity	0.962	0.934	0.964	0.939	0.944
Map resolution (Å), 0.143 FSC	3.11	3.14	3.45	3.49	3.44
Model resolution (Å), 0.5 FSC	3.2	3.3	3.5	3.6	3.6
Map sharpening *B*-factor (Å^2)	−80.83	−62.49	−102.615	−118.77	−92.616
Map versus model cross-correlation (CCmask)	0.76	0.79	0.81	0.77	0.81
**Model composition**					
No. non-hydrogen atoms	30143	29410	32464	29417	28283
No. protein residues	3872	3792	4025	3682	3484
No. nucleotide residues	0	0	53	53	53
Ligands	3	3	3	3	3
**Average *B*-factors (Å^2)**					
Protein	36.70	45.71	30.40	39.71	45.94
Nucleotide	-	-	107.79	90.12	112.41
Ligand	64.90	68.34	45.98	39.16	58.10
Water	-	-	-	-	-
**R.M.S. deviations from ideal**					
Bond lengths (Å)	0.002	0.002	0.002	0.002	0.002
Bond angles (°)	0.496	0.502	0.502	0.493	0.519
**Validation**					
MolProbity score	1.14	1.41	1.25	1.37	1.14
All-atom clashscore	3.53	4.39	3.54	4.03	3.45
Poor rotamers (%)	0.03	0.00	0.05	0.22	0.23
**Ramachandran plot**					
Favored (%)	98.41	96.82	97.48	96.97	98.01
Allowed (%)	1.59	3.18	2.52	3.03	1.99
Outliers (%)	0.00	0.00	0.00	0.00	0.00

**Name of structure**	**HelD$_{N\text{-}term}$-RP2**	**σ$^{A}_{N\text{-}helix}$-RP2**	**RPo**
PDB ID	**8R6P**	**8R6R**	**8QTI**
EMDB ID	**EMD-18956**	**EMD-18959**	**EMD-18650**
**Data collection and processing**			
Microscope	FEI Titan Krios		
Voltage (kV)	300		
Camera	Gatan K3 BioQuantum		
Magnification	105,000 x		
Nominal defocus range (μm)	0.5-2.9		
Exposure time (s)	2		
Electron exposure (e–/Å^2)	40		
Number of frames collected (no.)	40		
Number of frames processed (no.)	26		
Pixel size (Å)	0.8336		
Micrographs (no.)	18,072		
Total particle images (no.)	5,910,700		
**Refinement**			
Particles per class (no.)	132,995	48,587	111,613
Sphericity	0.953	0.967	0.823
Map resolution (Å), 0.143 FSC	3.16	3.89	3.09
Model resolution (Å), 0.5 FSC	3.3	4.0	3.2
Map sharpening *B*-factor (Å^2)	−111.804	−97.0678	−80.1484
Map versus model cross-correlation (CCmask)	0.77	0.70	0.82
**Model composition**			
No. non-hydrogen atoms	28732	27630	27858
No. protein residues	3458	3323	3329
No. nucleotide residues	85	84	93
Ligands	3	3	3
**Average *B*-factors (Å^2)**			
Protein	46.35	24.65	35.87
Nucleotide	168.89	106.32	86.95
Ligand	76.14	12.60	42.04
Water	-	-	-
**R.M.S. deviations from ideal**			
Bond lengths (Å)	0.002	0.002	0.002
Bond angles (°)	0.499	0.511	0.492
**Validation**			
MolProbity score	1.12	1.49	1.35
All-atom clashscore	3.24	4.70	3.44
Poor rotamers (%)	0.12	0.12	0.09
**Ramachandran plot**			
Favored (%)	98.05	96.34	96.71
Allowed (%)	1.95	3.66	3.29
Outliers (%)	0.00	0.00	0.00

Supplementary References:

1 Zheng, S. Q. *et al.* MotionCor2: anisotropic correction of beam-induced motion for improved cryo-electron microscopy. *Nature methods* **14**, 331-332 (2017). https://doi.org:10.1038/nmeth.4193

2 Rosenthal, P. B. & Henderson, R. Optimal determination of particle orientation, absolute hand, and contrast loss in single-particle electron cryomicroscopy. *Journal of molecular biology* **333**, 721-745 (2003). https://doi.org:10.1016/j.jmb.2003.07.013

3 Tan, Y. Z. *et al.* Addressing preferred specimen orientation in single-particle cryo-EM through tilting. *Nature methods* **14**, 793-796 (2017). https://doi.org:10.1038/nmeth.4347

4 Kimanius, D., Dong, L., Sharov, G., Nakane, T. & Scheres, S. H. W. New tools for automated cryo-EM single-particle analysis in RELION-4.0. *Biochem J* **478**, 4169-4185 (2021). https://doi.org:10.1042/BCJ20210708

5 Jakobi, A. J., Wilmanns, M. & Sachse, C. Model-based local density sharpening of cryo-EM maps. *eLife* **6** (2017). https://doi.org:10.7554/eLife.27131

6 Ramlaul, K., Palmer, C. M. & Aylett, C. H. S. A Local Agreement Filtering Algorithm for Transmission EM Reconstructions. *Journal of structural biology* **205**, 30-40 (2019). https://doi.org:10.1016/j.jsb.2018.11.011

7 Kouba, T. *et al.* The Core and Holoenzyme Forms of RNA Polymerase from Mycobacterium smegmatis. *J Bacteriol* **201** (2019). https://doi.org:10.1128/JB.00583-18

8 Hubin, E. A. *et al.* Structure and function of the mycobacterial transcription initiation complex with the essential regulator RbpA. *eLife* **6** (2017). https://doi.org:10.7554/eLife.22520

9 Boyaci, H., Chen, J., Jansen, R., Darst, S. A. & Campbell, E. A. Structures of an RNA polymerase promoter melting intermediate elucidate DNA unwinding. *Nature* **565**, 382-385 (2019). https://doi.org:10.1038/s41586-018-0840-5
