# Supplementary material for: Mycobacterial HelD connects RNA polymerase recycling with transcription initiation
Source: Nat Commun. 2024 Oct 9;15:8740. doi: 10.1038/s41467-024-52891-5 (PMC11464796; doi:10.1038/s41467-024-52891-5)
Supplement: Supplementary file 3 — Description Of Additional Supplementary File [file 41467_2024_52891_MOESM3_ESM.pdf]

Description of Additional supplementary files

Supplementary Movie 1

Description: Assembly of Msm HelD-σ A -RbpA-RNAP complex in State I and transition to State II

Supplementary Movie 2

Description: Assembly of Msm us-fork promoter-HelDσ A -RNAP complex in State II and transition to State III

Supplementary Movie 3

Description: Assembly of Msm HelDN-term-σ A -RNAP RP2-like complex upon binding of transcription bubble DNA and transition to RPo.

Supplementary Movie 4

Description: HelD release from RNAP All transitions between states were created by the morph algorithm in PyMOL. The event succession in the movies is based on the model proposed in Figure 6.
