# Supplementary material for: Mycobacterial HelD connects RNA polymerase recycling with transcription initiation
Source: Nat Commun. 2024 Oct 9;15:8740. doi: 10.1038/s41467-024-52891-5 (PMC11464796; doi:10.1038/s41467-024-52891-5)
Supplement: Supplementary file 8 — Reporting summary [file 41467_2024_52891_MOESM8_ESM.pdf]

nature portfolio

Corresponding author(s): Tomas Kouba, Libor Krasny, Jan Dohnalek

Last updated by author(s): Jun 10, 2024

Reporting Summary

Nature Portfolio wishes to improve the reproducibility of the work that we publish. This form provides structure for consistency and transparency in reporting. For further information on Nature Portfolio policies, see our Editorial Policies and the Editorial Policy Checklist.

Statistics

For all statistical analyses, confirm that the following items are present in the figure legend, table legend, main text, or Methods section.

n/a	Confirmed	
☐	☒	The exact sample size (*n*) for each experimental group/condition, given as a discrete number and unit of measurement
☐	☒	A statement on whether measurements were taken from distinct samples or whether the same sample was measured repeatedly
☐	☒	The statistical test(s) used AND whether they are one- or two-sided<br>*Only common tests should be described solely by name; describe more complex techniques in the Methods section.*
☒	☐	A description of all covariates tested
☒	☐	A description of any assumptions or corrections, such as tests of normality and adjustment for multiple comparisons
☐	☒	A full description of the statistical parameters including central tendency (e.g. means) or other basic estimates (e.g. regression coefficient) AND variation (e.g. standard deviation) or associated estimates of uncertainty (e.g. confidence intervals)
☐	☒	For null hypothesis testing, the test statistic (e.g. F, t, r) with confidence intervals, effect sizes, degrees of freedom and P value noted<br>*Give P values as exact values whenever suitable.*
☒	☐	For Bayesian analysis, information on the choice of priors and Markov chain Monte Carlo settings
☒	☐	For hierarchical and complex designs, identification of the appropriate level for tests and full reporting of outcomes
☒	☐	Estimates of effect sizes (e.g. Cohen's *d*, Pearson's *r*), indicating how they were calculated

Our web collection on statistics for biologists contains articles on many of the points above.

Software and code

Policy information about availability of computer code

Data collection	SerialEM 4.1, EPU 2.13,
Data analysis	MotionCor2 v1.3.1, CTFFIND 4.1.,TOPAZ v0.2.5, RELION 4.0, 3D FSC v 3.0, cryoEF v1.1.0, LAFTER, LocScale 0.1, Chimera 1.17.2, PyMol 3.0.2, CCP-EM 1.5, Coot 0.9.8.92, Molrep 11.7.02, MaxQuant version 1.6.3.4, Perseus version 1.6.2.3, SigmaPlot 8.0, WinCoot-0.8.9.2, Phenix program suite 1.19.2-4158, UCSF ChimeraX 1.6.1, ISOLDE 1.6.0, DNATCO 3.2, GraphPad Prism 7.02,PDBePISA 1.48, Office 365 v23-24, ggplot2 in R (R Core Team, 2022).

For manuscripts utilizing custom algorithms or software that are central to the research but not yet described in published literature, software must be made available to editors and reviewers. We strongly encourage code deposition in a community repository (e.g. GitHub). See the Nature Portfolio guidelines for submitting code & software for further information.

Data

Policy information about availability of data

All manuscripts must include a data availability statement. This statement should provide the following information, where applicable:

- Accession codes, unique identifiers, or web links for publicly available datasets
- A description of any restrictions on data availability
- For clinical datasets or third party data, please ensure that the statement adheres to our policy

Data Availability:

Atomic models coordinates and cryo-EM maps have been deposited in wwPDB and EMDB:
Msm HelD-σA-RbpA-RNAP complex State I :
EMD-18128 [https://www.ebi.ac.uk/pdbe/entry/emdb/EMD-18128]
PDB ID 8Q3I [http://doi.org/10.2210/pdb8Q3I/pdb]
Msm HelD-σA-RbpA-RNAP complex State II:
EMD-18511 [https://www.ebi.ac.uk/pdbe/entry/emdb/EMD-18511]
PDB ID 8QN8 [http://doi.org/10.2210/pdb8QN8/pdb]
Msm us-fork promoter-HelD-σA-RNAP complex State II:
EMD-18656 [https://www.ebi.ac.uk/pdbe/entry/emdb/EMD-18656]
PDB ID 8QU6 [http://doi.org/10.2210/pdb8QU6/pdb]
Msm us-fork promoter-HelD-σA-RNAP complex State III:
EMD-18873 [https://www.ebi.ac.uk/pdbe/entry/emdb/EMD-18873]
PDB ID 8R3M [http://doi.org/10.2210/pdb8R3M/pdb]
Msm us-fork promoter-HelDN-term-σA-RNAP complex State III:
EMD-18851 [https://www.ebi.ac.uk/pdbe/entry/emdb/EMD-18851]
PDB ID 8R2M [http://doi.org/10.2210/pdb8R2M/pdb]
Msm HelDN-term-σA-RNAP complex RP2-like:
EMD-18956 [https://www.ebi.ac.uk/pdbe/entry/emdb/EMD-18956]
PDB ID 8R6P [http://doi.org/10.2210/pdb8R6P/pdb]
Msm σAN-helix-RNAP complex RP2-like:
EMD-18959 [https://www.ebi.ac.uk/pdbe/entry/emdb/EMD-18959]
PDB ID 8R6R [http://doi.org/10.2210/pdb8R6R/pdb]
Msm RNAP open complex:
EMD-18650 [https://www.ebi.ac.uk/pdbe/entry/emdb/EMD-18650]
PDB ID 8QTI [http://doi.org/10.2210/pdb8QTI/pdb]

The mass spectrometry proteomics data are available in the ProteomeXchange Consortium via the PRIDE partner repository with the dataset identifier PXD046632 [doi:10.6019/PXD046632].

PDB accession codes of additional structures used in this study:
7PP4 [http://doi.org/10.2210/pdb7PP4/pdb]
5TW1 [http://doi.org/10.2210/pdb5TW1/pdb]
5VI5 [http://doi.org/10.2210/pdb5VI5/pdb]
6EE8 [http://doi.org/10.2210/pdb6EE8/pdb]
4XLS [http://doi.org/10.2210/pdb4XLS/pdb]
6YXU [http://doi.org/10.2210/pdb6YXU/pdb]
6YYS [http://doi.org/10.2210/pdb6YYS/pdb]
5VI8 [http://doi.org/10.2210/pdb5VI8/pdb]
5UHC [https://doi.org/10.2210/pdb5UHC/pdb]

Source data are provided with this paper.

Research involving human participants, their data, or biological material

Policy information about studies with human participants or human data. See also policy information about sex, gender (identity/presentation), and sexual orientation and race, ethnicity and racism.

Reporting on sex and gender	not applicable
Reporting on race, ethnicity, or other socially relevant groupings	not applicable
Population characteristics	not applicable
Recruitment	not applicable
Ethics oversight	not applicable

Note that full information on the approval of the study protocol must also be provided in the manuscript.

Field-specific reporting

Please select the one below that is the best fit for your research. If you are not sure, read the appropriate sections before making your selection.

☒ Life sciences ☐ Behavioural & social sciences ☐ Ecological, evolutionary & environmental sciences

For a reference copy of the document with all sections, see nature.com/documents/nr-reporting-summary-flat.pdf

Life sciences study design

All studies must disclose on these points even when the disclosure is negative.

Sample size	For biochemical analyses, sample sizes of at least of 3 were chosen (biological replicates) to provide significant results.
Data exclusions	No data were excluded.
Replication	Experiments were performed at least 3x (biological replicates). Reported data show individual replicates, means, and respective SD.
Randomization	not applicable
Blinding	not applicable

Reporting for specific materials, systems and methods

We require information from authors about some types of materials, experimental systems and methods used in many studies. Here, indicate whether each material, system or method listed is relevant to your study. If you are not sure if a list item applies to your research, read the appropriate section before selecting a response.

Materials & experimental systems

n/a	Involved in the study
☐	☒ Antibodies
☒	☐ Eukaryotic cell lines
☒	☐ Palaeontology and archaeology
☒	☐ Animals and other organisms
☒	☐ Clinical data
☒	☐ Dual use research of concern
☒	☐ Plants

Methods

n/a	Involved in the study
☒	☐ ChIP-seq
☒	☐ Flow cytometry
☒	☐ MRI-based neuroimaging

Antibodies

Antibodies used	ANTI-FLAG M2 Affinity Gel (cat. no. A2220)
Validation	https://www.sigmaaldrich.com/CZ/en/product/sigma/a2220

Plants

Seed stocks	not applicable
Novel plant genotypes	not applicable
Authentication	not applicable
